# Supplementary figures and images for: The proportion of core species in a community varies with spatial scale and environmental heterogeneity
Source: PeerJ. 2018 Nov 30;6:e6019. doi: 10.7717/peerj.6019 (PMC6276595; doi:10.7717/peerj.6019)

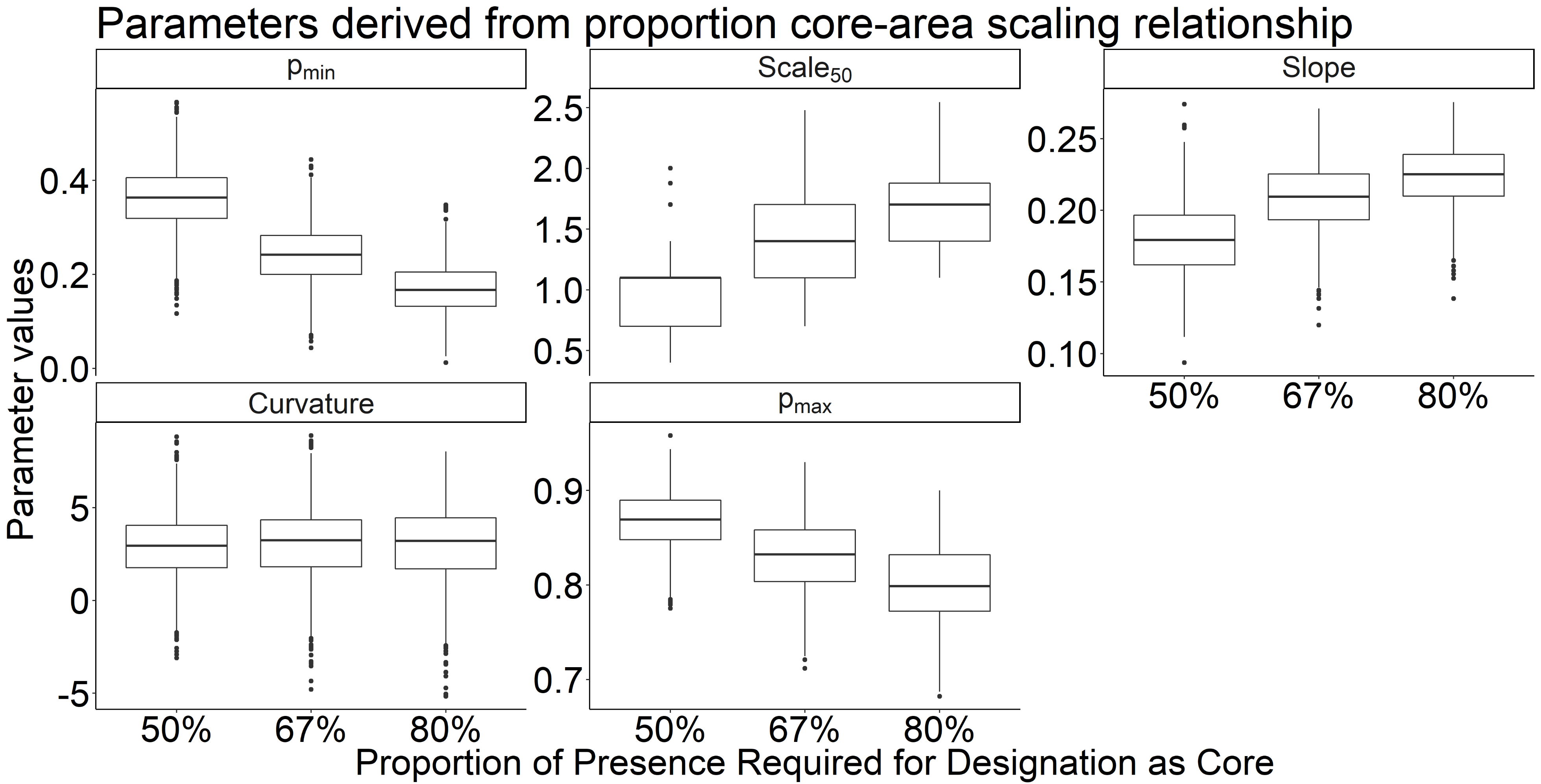

Supplement: Figure S1 — While there was not an appreciable difference between cutoffs, slight differences may indicate the resilience of higher cutoff thresholds against classification errors. [file peerj-06-6019-s001.png]

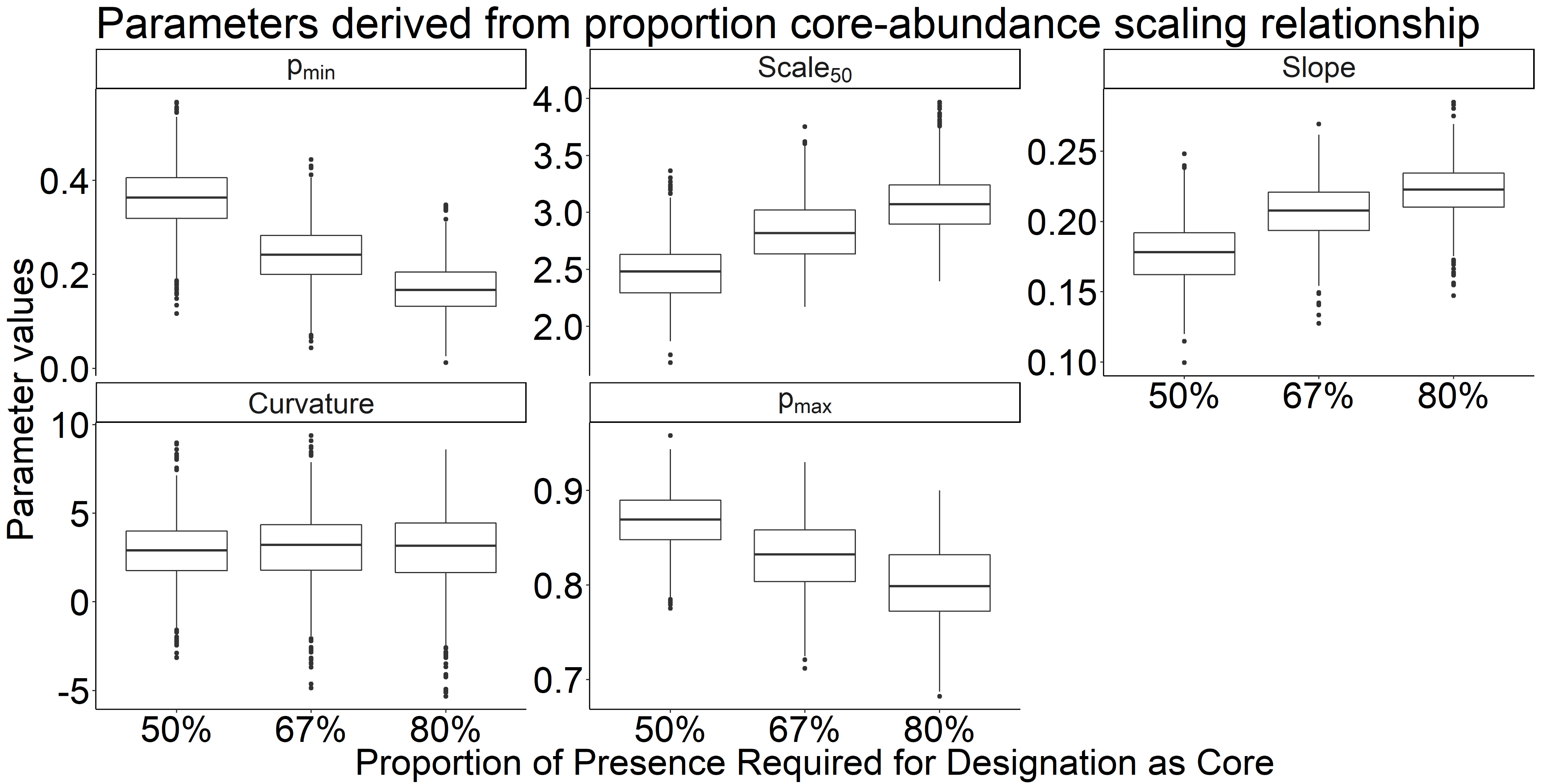

Supplement: Figure S2 — While there was not an appreciable difference between cutoffs, slight differences may indicate the resilience of higher cutoff thresholds against classification errors. [file peerj-06-6019-s002.png]

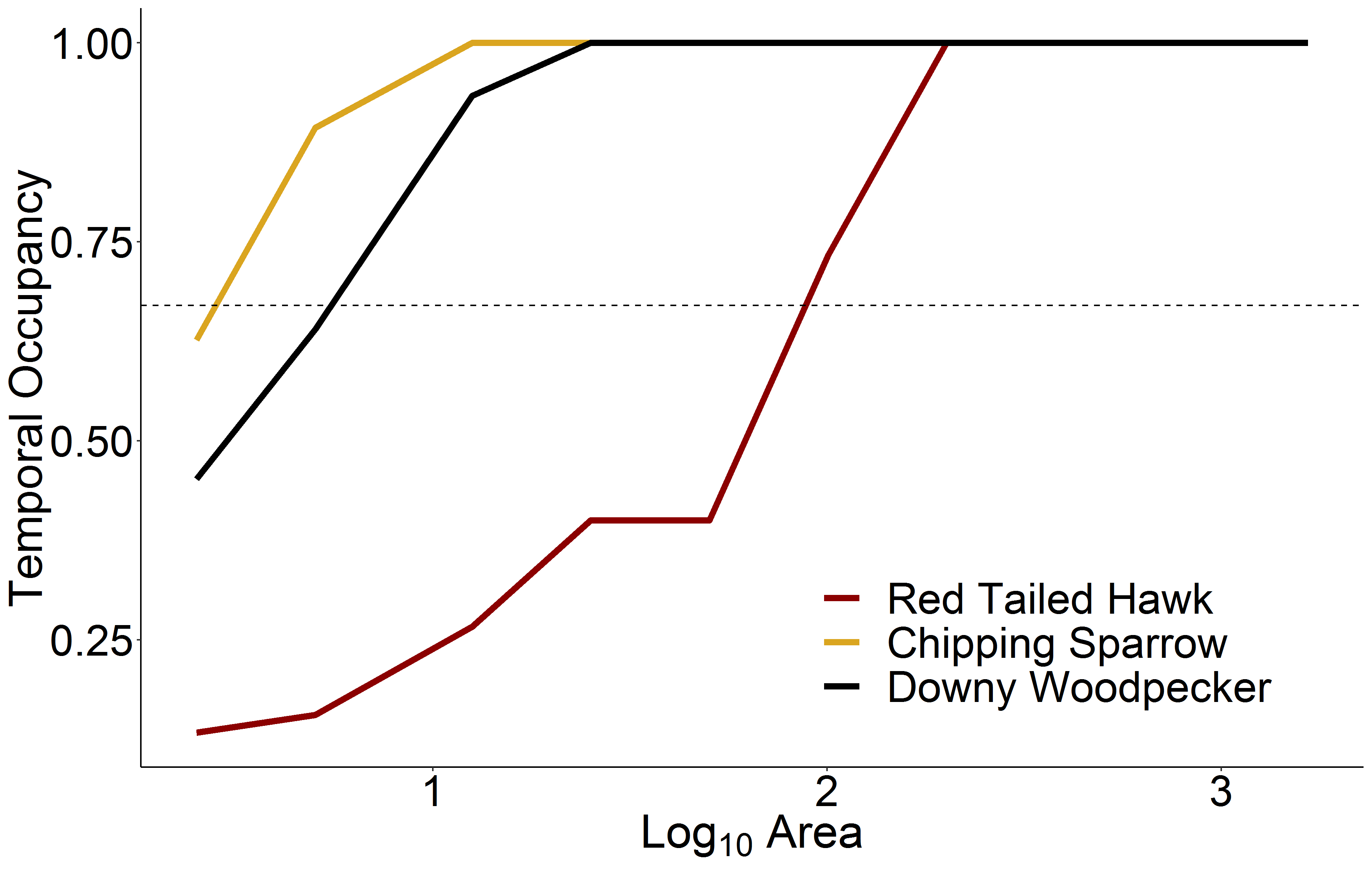

Supplement: Figure S3 — Temporal occupancy of three distinct avian species across scales, originating from a single focal route in Alabama (route 2001). Each species has different breeding territory space requirements (e.g., the Red Tailed Hawk requires large territories compared to a Chipping Sparrow, or an intermediate species like the Downy Woodpecker). These space requirements and differences in species ecology result in predictable differences in each species temporal occupancy, namely that species with large territory requirements will become core species at larger scales than species with smaller territory requirements. [file peerj-06-6019-s003.png]
